# A comparative investigation between ProTaper Next, Hyflex CM, 2Shape, and TF-Adaptive file systems concerning cyclic fatigue resistance

**DOI:** 10.34172/joddd.2021.029

**Published:** 2021-08-25

**Authors:** Sibel Koçak, Faruk Furkan Şahin, Olcay Özdemir, Mustafa Murat Koçak, Baran Can Sağlam

**Affiliations:** ^1^Department of Endodontics, Faculty of Dentistry, Zonguldak Bülent Ecevit University, Zonguldak, Turkey; ^2^Private Dental Clinic, Istanbul, Turkey; ^3^Department of Pedododontics, Faculty of Dentistry, Zonguldak Bülent Ecevit University, Zonguldak, Turkey

**Keywords:** 2Shape, Hyflex CM, ProTaper Next, Scanning electron microscope, Static cyclic fatigue, TF-Adaptive

## Abstract

**Background.** This study aimed to compare the cyclic fatigue resistance of ProTaper Next, Hyflex CM, 2Shape, and TF-Adaptive nickel-titanium endodontic file systems with various alloy properties and production methods and investigate the fractured cross-sectional surface of files due to cyclic fatigue by scanning electron microscopy (SEM).

**Methods.** A total of 120 instruments were used (n=30). For standardization, #25/.06 apical diameter and taper angle were selected for each file system. The experiment of files was subjected to a static cyclic fatigue model. The time for files’ failure was recorded with a digital chronometer and multiplied by the rotation speed to calculate the number of cycles. Kolmogorov-Smirnov, one-way ANOVA, and post hoc Bonferroni analysis were used for statistical analysis. Statistical significance was set at *P* < 0.05.

**Results.** The number of cycles for the failure of files was compared between the groups, and significant differences were found (*P* < 0.05). The number of cycles for instrument failure was recorded from the highest to the lowest as follows: Hyflex CM, TF-Adaptive, ProTaper Next, and 2Shape.

**Conclusion.** The files were fractured at different average numbers of cycles in an artificial canal in all the groups. The Hyflex CM demonstrated better cyclic fatigue resistance than TF Adaptive, ProTaper Next, and 2Shape file systems. Factors such as production patterns, alloy properties, and the phase in which the files were produced might affect the lifespan of file systems.

## Introduction

Nickel-titanium (NiTi) rotary files have gain popularity for preparing and shaping the root canal system because of their cutting capacity, elasticity, and efficiency.^1^ Complications such as ledges, zip perforation, and straightened root canals occur less frequently using NiTi rotary files.^2^ Although NiTi files have many advantages, they present a risk of failure in curved or S-shaped canals, which might adversely affect treatment prognosis.^3^ However, many factors that play a role in NiTi files’ failures, such as cyclic and torsional fatigue, are the most critical reasons.^4^ The operational speed, motion principle, and metallurgic and surface characteristics are substantial factors that can cause cyclic fatigue of instruments.^5^

Different cross-sectional designs and alloys have been proposed to resist fatigue failure of rotary files and increase their flexibility.^1^ Various thermally treated NiTi alloys, such as CM-Wire, M-Wire, T-Wire, and R-phase, have been introduced for optimizing the transformation behavior of NiTi alloy and microstructure, which affect the mechanical structure.^1,6^

ProTaper Next (PTN, Dentsply Tulsa Dental Specialties, Tulsa, OK) has a rectangular cross-section and a variable taper that offsets the center point. It is manufactured of M-wire alloy that underwent a specific thermomechanical process to improve cyclic fatigue resistance.^7^

HyFlex CM (HCM, Coltene/Whaledent, Inc, Cuyahoga Falls, OH, USA), manufactured of CM-Wire, is a shape memory Ni-Ti file termed ‘controlled memory.’ This file system is made by a lower percentage of nickel weight than most commercially available Ni-Ti, as claimed by the manufacturer.^8^ Because of its content and proprietary manufacturing process, HCM does not rebound to its original shape, unlike conventional Ni-Ti file systems. Their higher flexibility might lead to a reduced risk of failure due to ledge formation, canal transportation, or perforation.^9^

2Shape (TS, Micro‐Mega, Besancon, France), manufactured using T-Wire alloy, is produced with proprietary heat treatment. It has been claimed that the manufacturing process aims to improve cyclic fatigue resistance.^10^

TF Adaptive (TFA, SybronEndo, Orange, CA, USA) was manufactured by a twisting method. It was claimed that TFA runs in the longitudinal direction because of its different natural grain structure by the manufacturer, and the file system is made of the R-phase of NiTi alloy and has a higher fracture resistance.^5^ Differently, TFA is used with both continuous rotation and reciprocation for combining the advantages of motions. The combined movement was called ‘adaptive motion’ by Axis, Sybron-Endo, automatically adapting to instrumentation stresses.^11^

NiTi alloy properties and production methods might influence the cyclic fatigue resistance of endodontic files.^1^ There is no comparison of the M-wire, CM-wire, T-wire, and R-phase NiTi alloy to the best of our knowledge. This study aimed to evaluate and compare ProTaper Next, Hyflex CM, 2Shape, and TF-Adaptive file systems made of different alloy properties (M-wire, CM-wire, T-wire, and R-phase) and production methods concerning cyclic fatigue resistance and investigate the fractured cross-sectional surface due to cyclic fatigue by scanning electron microscopy (SEM).

## Methods

Thirty PTN X2 (#25, 0.06 variable taper, 300 rpm, 2 Ncm), 30 HCM (#25, 0.06 continuous taper, 500 rpm, 2.5 Ncm), 30 TS2 (#25, 0.06 continuous taper, 350 rpm, 2 Ncm), and 30 TFA SM-2 (#25, 0.06 continuous taper, TF-Adaptive 8:1 mode) were selected. All the samples were inspected for any defect or irregularity with the naked eye and under a stereomicroscope (Olympus SZ61 stereomicroscope: Olympus, Tokyo, Japan) at ×20 magnification. The instruments demonstrating any deformity were excluded.

The files were subjected to a static cyclic fatigue model similar to previous studies.^12^ The artificial root canal was manufactured compatible with the instrument’s dimensions. Synthetic oil was used within the root canal to reduce friction between the canal’s metallic walls and the file. The mechanism rotated freely inside an artificial canal with a 60° angle of curvature, 5 mm radius, and 1.5 mm of inner diameter.

All the systems were rotated in the artificial canal at room temperature, and a 1/100-second chronometer was used to record the time for failure. The stainless steel artificial canal had a glass window so that failure was easily detectable. The time for the failure of files was recorded with a digital chronometer and multiplied by the rotation speed to calculate the number of cycles.

SPSS 19.0 (SPSS Inc, Chicago, IL, USA) was used for statistical analysis. The data were analyzed using the Kolmogorov-Smirnov test to verify the assumption of normality. One-way ANOVA was used to compare the data in the normal distribution, and post hoc Bonferroni analysis was used for the difference between the groups. A significance level of 0.05 indicates a 5% risk of concluding that a difference exists when there is no actual difference.

## Results

**Figure 1:**
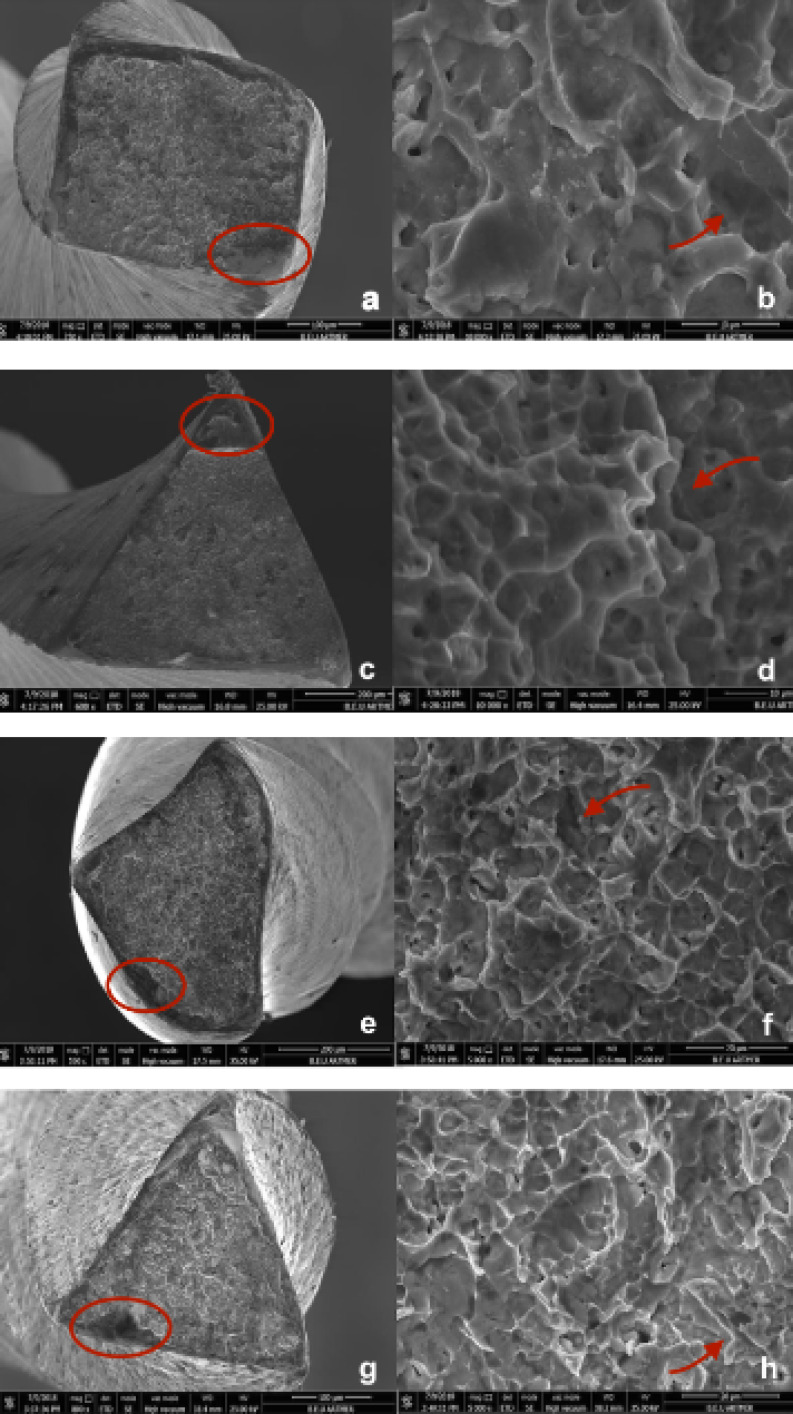
Representative fractured cross-sectional surface SEM images of the tested files. The red circles present a magnification area, and the red arrows show abrupt breakage (a. PTN ×750, b. PTN ×10000, c. HCM ×600, d. HCM ×10000, e. TS ×550, f. TS ×5000, g. TFA ×800, h. TFA ×5000).

Table 1 presents the means and standard deviations of the number of cycles until fracture occurred and the lengths of fractured segments. According to the statistical analysis, the HCM file (1221.7±47) had the highest, and the TS file (291±46) had the least fatigue resistance (P < 0.05). The fractured lengths of the files were recorded, and the PTN (3,85±0.25) demonstrated a significant difference compared to other groups (*P* < 0.05). There was no significant difference in the mean length of the fractured fragments between HCM (4.5±0.35), TS (4.3±0.26), and TFA (4.31±0.34)systems (*P* > 0.05). Figure 1 presents the representative SEM images of the fractured cross-sectional surface of all the groups.

**Table 1:** The means and standard deviations (SD) of the number of cycles until fracture occurred and the lengths of fractured segments (*P* < 0.05)

**Ni-Ti Files**	**Number of cycles**	**SD**	**Lengths**	**SD**
PTN	554.7^a^	59	3.85^x^	0.25
HCM	1221.7^b^	47	4.5^y^	0.35
TS	291^c^	46	4.3^y^	0.26
TFA	798.8^d^	57	4.31^y^	0.34

*Note*. Different superscript letters indicate statistically significant differences between groups (*P* < 0.001).

## Discussion

The cyclic fatigue test aims to examine and determine the purely physical properties of NiTi files. Although the extracted tooth model better mimics the clinical conditions, it cannot be used as an ideal model. In this study, the extracted tooth model was not used to provide a standard model and minimize the contribution of parameters other than cyclic fatigue.

According to previous studies, file systems’ cyclic fatigue resistance has been subjected to various dynamic and static models.^13,14^ In static cyclic fatigue tests, the files are used in a fixed working length and rotated until a fracture occurs.^15^ Dynamic test devices include axial movements in addition to the static model.^16^ Gavini et al^17^ investigated the importance of selecting the test model correctly for the experimental setup and reported that the dynamic model has limitations compared to the static model. The static model provides a clear pathway in the artificial canal, and horizontal movement is entirely subjective as it is controlled manually. An advantage is that the results are consistent and reproducible with clinical practice.^12^ Due to its consistency and reproducibility, the static model was used in this study. It aimed to place each NiTi file correctly in the artificial canal and simultaneously position it in three dimensions.

Although artificial canals with various internal diameters and curvature angels have been used in previous studies, limitations have also been considered.^12,18^ A 60° angle of curvature, 5-mm radius, and 1.5-mm inner diameter of a stainless steel artificial canal designed by Pruett et al^18^ was used in this study. The curvature was located at the 5-mm apical tip of the artificial root canal. There was no force on the file in the coronal part, which did not affect the working time. In this way, all the files were used in the canal with the same curvature angle, and the effects of the physical properties of the files on cyclic fatigue were investigated.

Haïkel et al^19^ reported that the taper angle and apical diameter might significantly change the operating time of the instruments. In this study, the taper angle and apical diameter of all the files used were kept similar to provide standardization.

To minimize file fracture, which is the most common complication in root canal preparation, manufacturers develop new generations of files with superior mechanical properties by improving metallurgy and production methods.^20^ The unique nanocrystalline microstructure of martensitic M-wire alloy, which is optimized by thermomechanical processes, aims to have more flexibility, more outstanding durability, and abrasion resistance to the files manufactured from traditional superelastic NiTi alloy.^21^ Previous studies have shown that M-wire alloy file systems are more resistant to cyclic fatigue than traditional file systems.^22-24^ The horizontal stress of files with the asymmetric design was reported to occur less than other file systems.^25^ The asymmetric design makes it possible for the file to be positioned outside the center of the root canal during the rotation movement, creating different stress points. Our study showed that the average number of cycles of the PTN file system was consistent with the studies mentioned above. However, we found that TS was the only file system to exhibit less resistance compared to PTN. This conflicting result might be explained by the flexibility and cyclic fatigue resistance of the M-wire alloy PTN file system. The stress of the file might decrease due to its snakelike movement along its long axis during rotational movements.

After strengthening by applying thermomechanical processes, files without shape memory were produced to increase cyclic fatigue resistance. The HCM file system was made from CM-wire alloy, which demonstrates a mixture of austenite and martensitic phases at room temperature, rather than austenite phase like conventional NiTi. The martensitic phase is softer than the austenite phase due to the low Young’s modulus, and the CM-wire file is mostly in the martensitic phase.^26^

Many studies have reported that the HCM is more flexible and exhibits higher cyclic fatigue resistance than other files with different properties and metallurgies.^27,28^ Topcuoğlu et al^29^ evaluated the cyclic fatigue resistance of HCM, ProTaper Universal (Dentsply Tulsa Dental Specialities, Tulsa, OK), PTN, and OneShape (Micro Mega, Besancon, France) file systems in an artificial canal with two curvature angels and reported that PTN and HCM file systems were more durable in the apical curvature than other groups. However, there was no statistically significant difference in coronal curvature. The results of the present study were compatible with previous results. The HCM was found to be more resistant to cyclic fatigue. The flexibility of the HCM file system has been enhanced because it does not have the shape memory feature. It is produced in the intermediate phase between the austenite and martensitic phases, which might be associated with the higher fatigue resistance.^30^

The TS file system was introduced in 2017 by heat treatment with T-Wire technology with an asymmetric design like PTN. Özyürek et al^10^ evaluated the cyclic fatigue resistance of Hyflex EDM (Coltene/Whaledent, Allstatten, Switzerland), Wave One Gold (Dentsply Maillefer, Ballaruies, Switzerland), Reciproc Blue (VDW, Munich, Germany), and TS file systems in artificial canals with two different curvature angles of 45° and 90°. The results showed that the Hyflex EDM file system was significantly more resistant than the TS file system. Besides, Pedullà et al^31^ evaluated cyclic fatigue resistance between HCM and TS and concluded that HCM had higher cyclic fatigue resistance than TS. Olcay et al^32^ investigated the cyclic fatigue resistance of the WaveOne Gold, PTN, and TS instruments. According to the results, PTN was more resistant than the TS file. In the present study, the results were consistent with previous studies. The HCM and PTN file systems were more resistant, with a higher than average number of cycles until fracture occurred than the TS group. A study evaluated 25/.04 taper of the file systems at an intracanal temperature, including 2Shape TS1, Twisted File (Axis/SybronEndo, Orange, CA), and EndoSequence Xpress (ESX; Brasseler, Savannah, USA) and showed that 2Shape had the highest cyclic fatigue-resistant compared to other systems.^33^ The conflicting results might be due to different tapers of the files and the ambient temperature. The TS files, which have an asymmetric design such as PTN, might have a low resistance to breakage caused by cyclic fatigue at room temperature due to the newly produced T-wire alloy. The cyclic fatigue resistance of the TS file might have reduced with a decrease in ambient temperature. Klymus et al^34^ and Dosanjh et al^35^ concluded that the temperature affected the cyclic fatigue of NiTi instruments. In this case, it might be crucial to control the irrigation solutions’ temperature when using a TS file.

In the present study, the HCM file system demonstrated more resistance than the TFA group. The TFA file system was more resistant than the PTN and TS group at 60° angle of curvature, 5 mm radius, and 1.5-mm inner diameter of artificial canals. Due to its flexibility, alloy properties, and production methods, the HCM file system is significantly more durable than other groups. The higher resistance of TFA files than the PTN and TS files might be explained by the difference in adaptive working principle and the minimization of defects on the file due to the R-phase’s bending procedures.

It is crucial to evaluate the rotary file systems to provide better cyclic fatigue resistance to minimize mechanical complications during endodontic treatment. Hyflex CM demonstrated comparable and promising results in cyclic fatigue resistance than TFA, PTN, and 2S files.

## Conclusion

The files were fractured at the different average number of cycles in an artificial canal in all groups. The Hyflex CM demonstrated better cyclic fatigue resistance than TF Adaptive, ProTaper Next, and 2Shape file systems. Factors such as production patterns, alloy properties, and the phase in which the files were produced might affect the file systems’ lifespan.

## Authors’ Contributions

All authors contributed to the study conception and design. Material preparation, data collection, and analysis were performed by FFŞ, SK, MMK and BCS. The first draft of the manuscript was written by OÖ, and all authors commented on previous versions of the manuscript. All authors read and approved the final manuscript.

## Funding

The study was supported by the Scientific Research and Development Department of Zonguldak Bülent Ecevit University (Grant number: 2017-27194235-04).

## Competing Interests

The authors declare that no conflict of interests.

## Ethics approval

Not applicable.
